# Interactive tool to create adjustable anatomical atlases for mouse brain imaging

**DOI:** 10.1007/s10334-020-00866-0

**Published:** 2020-07-21

**Authors:** Markus Sack, Lei Zheng, Natalia Gass, Gabriele Ende, Alexander Sartorius, Wolfgang Weber-Fahr

**Affiliations:** 1 Department of Neuroimaging, Central Institute of Mental Health (CIMH), Medical Faculty Mannheim, University of Heidelberg, Mannheim, Germany; 2 Research Group Translational Imaging, Central Institute of Mental Health (CIMH), Medical Faculty Mannheim, University of Heidelberg, Mannheim, Germany; 3 Data Analysis and Modeling in Medicine, Mannheim Institute for Intelligent Systems in Medicine, Medical Faculty Mannheim, Heidelberg University, Mannheim, Germany; 4 Department of Psychiatry and Psychotherapy, Central Institute of Mental Health (CIMH), Medical Faculty Mannheim, University of Heidelberg, Mannheim, Germany

**Keywords:** MRI, Mouse brain atlas, Tool, Atlas creation, Imaging

## Abstract

**Objective:**

Brain atlases are important research tools enabling researchers to focus their investigations on specific anatomically defined brain regions and are used in many MRI applications, e.g. in fMRI, morphometry, whole brain spectroscopy, et cetera. Despite their extensive use and numerous versions they usually consist of predefined rigid brain regions with a given level of detail often degrading them to a non-ideal tool in special research topics.

**Result:**

To overcome this intrinsic weakness we present a graphical user interface application which allows researchers to easily create mouse brain atlases with an adjustable user-defined level of detail and coverage to match specific research questions.

## Introduction

In many Magnetic Resonance Imaging (MRI) post-processing procedures brain atlases are used to investigate anatomically defined brain regions like e.g., in functional-MRI, morphometry, whole brain spectroscopy, et cetera. These brain atlases enable researchers to focus their investigations on specific brain areas by combining the voxel-based MR data from one or several brain regions of interest (ROI).

The main limitation of the currently available brain atlases standardly used in MR research (for instance WFU Pickatlas [1], AAL [2], Talairach [3], Dorr [4]) is their inflexibility due to an initial ROI pre-definition. The modification of pre-defined brain regions can be complicated and, thus, the level of detail is fixed. For instance, defining a sub-area within a ROI is usually only possible with considerable effort and requires expert knowledge.

An MRI atlas usually consists of two datasets: 1) an annotation image file which comprises integer numbers in a 3D space, representing the location of anatomically defined brain regions, and 2) a corresponding file (often a text file) which links the numbers of the annotation image with the name of the brain regions.

## Material and method

We present a GUI (graphical user interface) application (written in MATLAB, The MathWorks, Inc., Natick, Massachusetts, United States) which allows researchers to easily create mouse brain atlases with an adjustable level of detail and coverage to match specific research questions.

Based on the Allen Mouse Common Coordinate Framework brain atlas [5] the user of our application can select and afterwards export ROIs in a newly created atlas. This procedure yields an image file (in nifty format;.nii) containing the annotation (anatomical) information together with a text file linking the integer numbers of the annotation image with the brain regions. These files can then be used like any other typical atlas-defining files.

The underlying data structure of the Allen Mouse brain atlas is initially given in big brain divisions (e.g., ‘Basic cell groups’, ‘fiber tracts’, etc.) as ‘parent’-regions whose children, grandchildren, great-grandchildren and so on are defining finer, and thus more specific, brain regions. Due to the high resolution of the Allen atlas (up to 10 µm; isotropic) ROIs can reach the level of single neuronal layers. As a compromise between data size and detail of ROIs, we chose the 50 µm variant.

Furthermore, for convenience the annotation image is additionally transformed into the Paxinos space by a SPM batch file, which could be replaced by experienced users (details described in the manual).

### Transformation into the Paxinos space

**Fig. 1:**
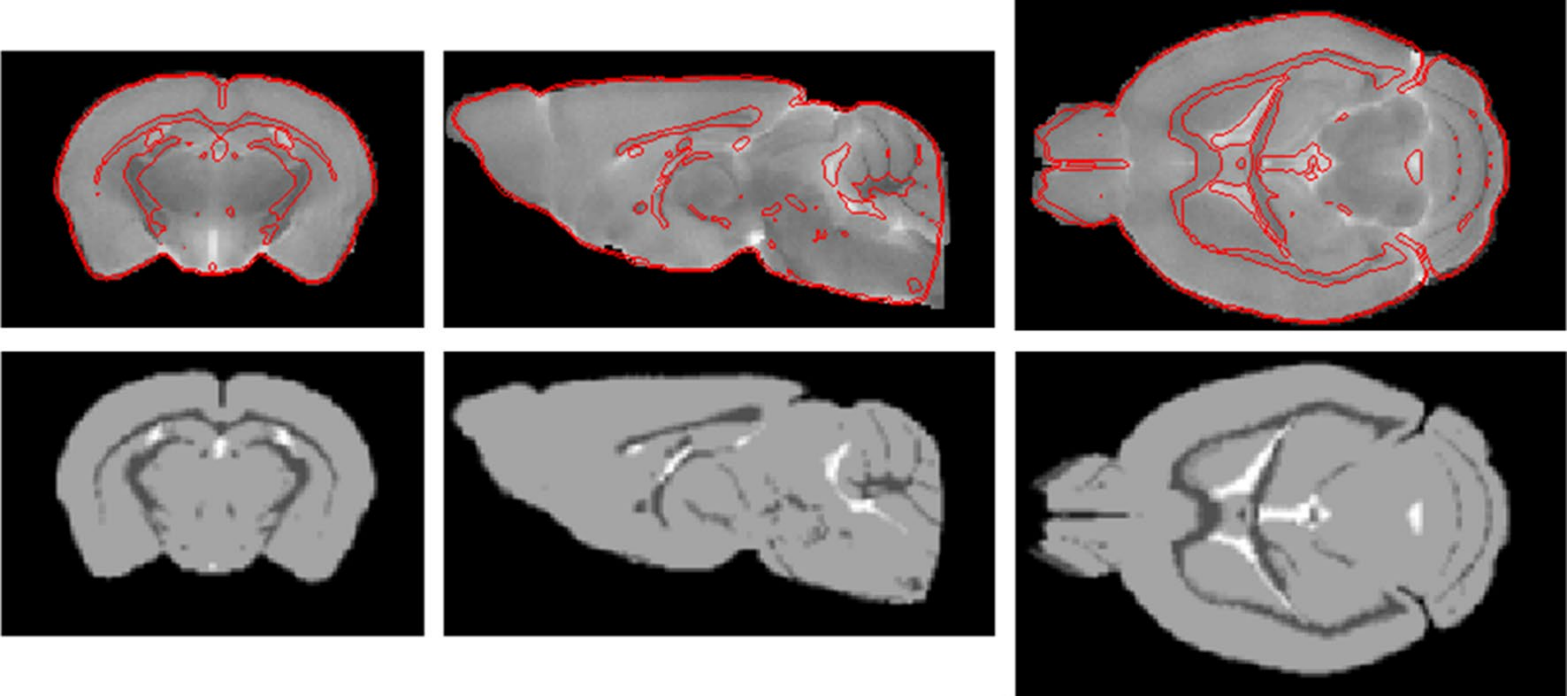
Upper row: Used anatomical template and contour plot of transformation result from ‘Allen’ to the Paxinos space. Lower row: Artificially created image as described in method section after transformation

To transform the Allen atlas data into the Paxinos space SPM’s ‘oldnormalize’ function was used with the anatomical/structural image from the Dorr atlas [4] as template, which was previously transformed into the Paxinos space [6, 7]. Since the provided average Nissl image of the Allen atlas was not suitable for a transformation with a satisfactory result (most probably due to different image modalities), we created a simulated anatomical image. To do so, we defined ROIs consisting solely of grey matter, white matter, and cerebrospinal fluid and assigned values to them mimicking the intensity distribution of the template, respectively (see Fig. 1 for results). The thus obtained transformation field is then applied to newly created atlases via an SPM batch file.

## Result

**Fig. 2:**
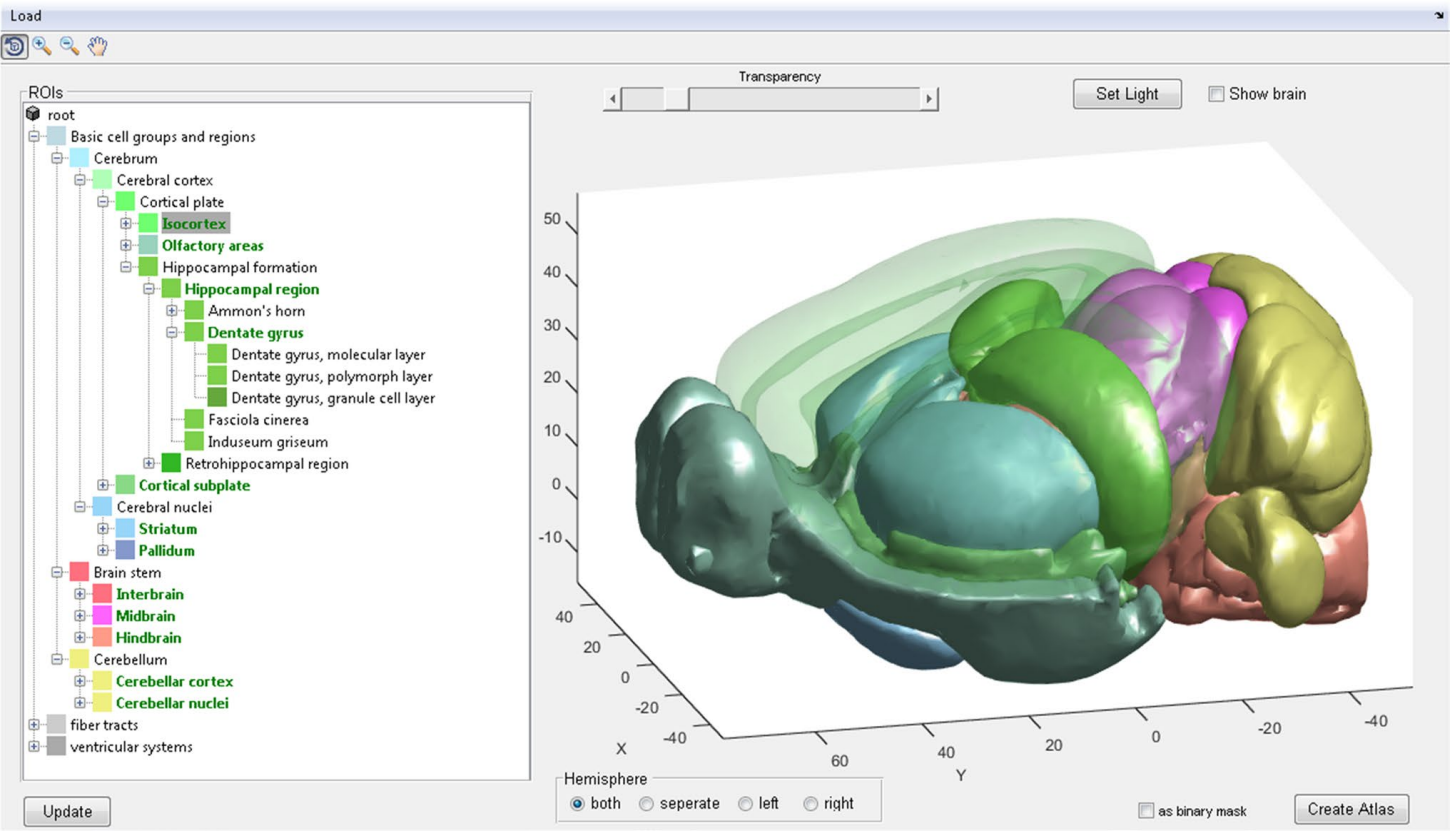
Application overview. On the left panel: an interactive tree showing an example of available ROIs. The selected ROIs are in green, bold font. On the right: a 3D illustration of selected ROIs colored corresponding to the ROI icons of the tree selection. For better visibility ROIs can be made transparent (here shown for the ‘Isocortex’ ROI)

**Fig. 3:**
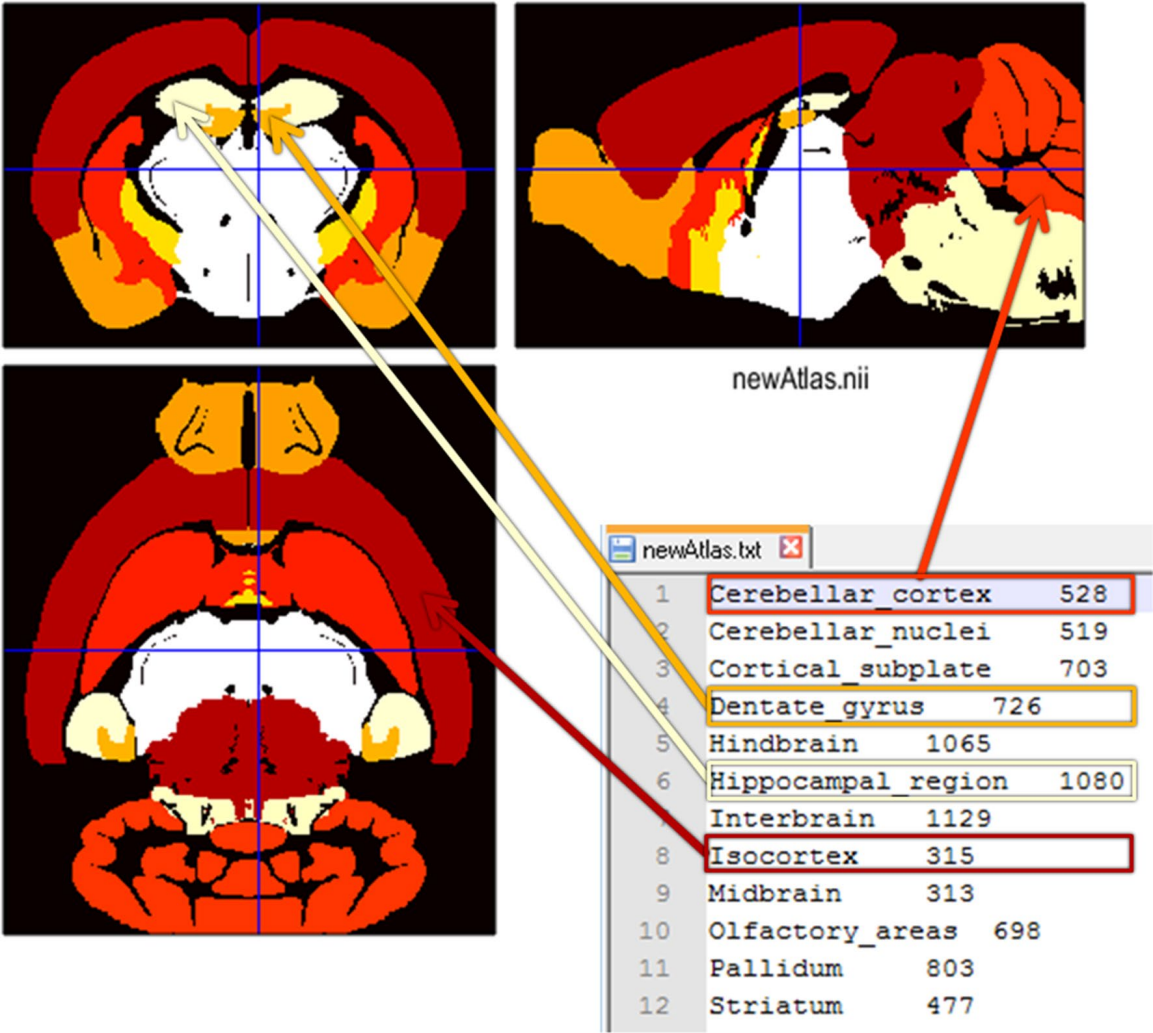
Exemplary created atlas in the Paxinos space with corresponding txt file linking the integer numbers of the annotation image file with the name of selected brain regions. Note that the parent structure ‘Hippocampal region’ and one of its sub-regions (child ‘Dentate gyrus’) are defined simultaneously

In the application, the ROI hierarchical data structure is represented as a tree (Fig. 2, left) which grows with the respective levels of detail. To select ROIs the user right-clicks on a node in the tree (selected regions are in a green font) and the application combines the subjacent defined brain structure. For a coarse visual inspection, a 3D model of the selected ROIs can be presented (the ‘transparency’ slider sets the transparency of a selected ROI, see Fig. 2, right). After creating an atlas, it is immediately presented in the SPM [8] ‘Check Reg’ function, providing the possibility for a more detailed inspection (Fig. 3; with an additional example of the annotation text file).

Further functionalities of our application include:

- Exporting selected ROIs as binary mask files each representing the selected ROIs.
- Selecting the hemisphere: ROIs can be separated in both, or in the left and right hemisphere.
- Possibility to select ‘parental’ ROIs and additionally some of their sub-areas.
- For an easier integration of already established post-processing routines, the created atlas is automatically transformed into the Paxinos space.
- A ‘load’ function based on a.txt file to modify an already created atlas.

The atlas can be used as any other conventional atlas dataset in structural, functional analysis workflows, e.g., in SPM’s ‘imcalc’ routine using an expression like ‘i1.*(i2 =  = XX)’ (where XX represents the ROI number), using a binary mask directly in FSL’s ‘fslmask’ routine, or even implement the atlas in other apps like the CONN toolbox (www.nitrc.org/projects/conn, RRID:SCR_009550) [9]. For further details the user is referred to the corresponding manuals of these tools. Furthermore, example scripts of ROI time course extraction are provided in the online repository.

## Discussion

The presented application allows scientists to create mouse brain atlases with a level of detail matching their obtained data resolution and/or their specific research question. Due to its simplicity, flexibility, and adaptability we hope that it becomes a useful tool. The application can be downloaded here: https://github.com/DrCarbonCIMH/MouseAtlas.

It should be noted that, as with any other tools, it is the user’s responsibility to take care about the correctness and accuracy of the created data, especially regarding ROI definition and space transformation.

Furthermore, we will explore the possibility to extend the application by other datasets such as developing mouse, rat, or human brain atlases.
